# Claudin-5: gatekeeper of neurological function

**DOI:** 10.1186/s12987-019-0123-z

**Published:** 2019-01-29

**Authors:** Chris Greene, Nicole Hanley, Matthew Campbell

**Affiliations:** Trinity College Dublin, Smurfit Institute of Genetics, Dublin 2, Ireland

**Keywords:** Blood–brain barrier, Endothelial cell, Tight junction, Claudin-5

## Abstract

Tight junction proteins of the blood–brain barrier are vital for maintaining integrity of endothelial cells lining brain blood vessels. The presence of these protein complexes in the space between endothelial cells creates a dynamic, highly regulated and restrictive microenvironment that is vital for neural homeostasis. By limiting paracellular diffusion of material between blood and brain, tight junction proteins provide a protective barrier preventing the passage of unwanted and potentially damaging material. Simultaneously, this protective barrier hinders the therapeutic effectiveness of central nervous system acting drugs with over 95% of small molecule therapeutics unable to bypass the blood–brain barrier. At the blood–brain barrier, claudin-5 is the most enriched tight junction protein and its dysfunction has been implicated in neurodegenerative disorders such as Alzheimer’s disease, neuroinflammatory disorders such as multiple sclerosis as well as psychiatric disorders including depression and schizophrenia. By regulating levels of claudin-5, it is possible to abrogate disease symptoms in many of these disorders. This review will give an overview of the blood–brain barrier and the role of tight junction complexes in maintaining blood–brain barrier integrity before focusing on the role of claudin-5 and its regulation in homeostatic and pathological conditions. We will also summarise therapeutic strategies to restore integrity of cerebral vessels by targeting tight junction protein complexes.

## Introduction

**Fig. 1:**
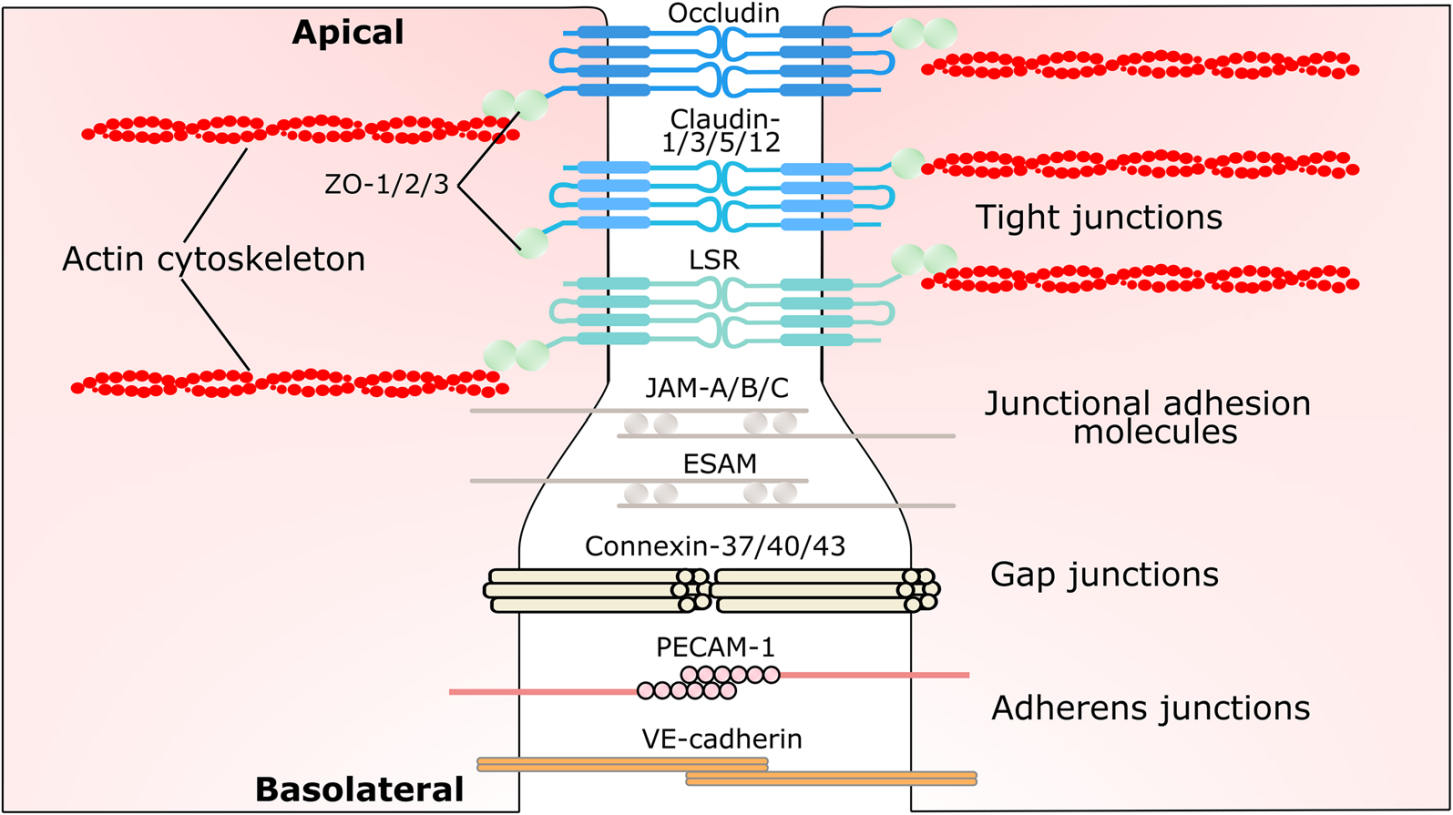
Inter-endothelial connections. Located closest to the apical membrane are the tight junction proteins consisting of claudin-1, -3, -5, -12; occludin; and lipolysis-stimulated protein (LSR) which limit paracellular diffusion of ions and solutes across the tightly packed monolayer of endothelial cells. Zonula occludens (ZO)-1, -2 and -3 binds to PDZ motifs on intracellular domains of claudins and occludin and bind to the actin cytoskeleton, providing structural integrity to the tight junction. Other junctional complexes contribute to tight junction properties including junctional adhesion molecule (JAM)-A, -B and -C and endothelial cell adhesion molecules (ESAM). Gap junctions such as connexin-37 and -40 form hemichannels between opposing endothelial cell membranes, contributing to intercellular communication. Located closest to the basolateral membrane are the adherens junction proteins including vascular endothelial (VE)-cadherin and platelet endothelial cell adhesion molecule-1 (PECAM-1)

The blood–brain barrier (BBB) is formed by a tightly packed monolayer of non fenestrated endothelial cells lining the brain capillaries which are enveloped by pericytes and perivascular astrocytes. The BBB forms a protective layer in the central nervous system (CNS) strictly limiting molecular exchange between the circulating blood and brain microenvironment. This is to ensure a constant state of homeostasis for efficient neural signalling. Accounting for just 2% of bodily mass, the brain and neuronal functions consume as much as 20% of the body’s oxygen and glucose needs despite a lack of energy reserves in the brain [1]. Therefore, blood vessels in the brain provide vital energy and nutrients in response to the metabolic demands of neurons (a process known as hyperaemia) [2]. Cerebral capillaries account for 85% of vessel length in the brain, providing a surface area of ~ 12 m^2^ of the endothelial surface for molecular exchange and an approximate 1:1 ratio of capillaries to neurons [3–5]. Owing to the restrictive regenerative capacities of neurons, there is a vital need to limit the unrestricted movement of material between the blood and brain and vice versa to prevent unwanted neuronal excitation. The BBB is formed by a monolayer of tightly packed, specialised endothelial cells along the cerebral vasculature that are mechanically linked together by tight junction protein complexes that eliminate the paracellular space between neighbouring endothelial cells. Additionally, the presence of numerous luminal and abluminal protein transporters, solute and ion transporters and efflux transporters strictly controls the entry and exit of molecules and metabolic by-products across cells along the transcellular pathway. This results in low paracellular and transcellular permeability. While endothelial cells form the BBB, it is the complex interactions of astrocytes, pericytes, neurons, microglia, and the acellular basement membrane forming the neurovascular unit, that are essential for the development of barrier properties, neurovascular coupling, angiogenesis and neurogenesis [6–8]. Tight junctions, located closest to the apical membrane, maintain BBB integrity by preventing the diffusion of proteins between luminal and abluminal membrane compartments and limit paracellular diffusion of ions and solutes across the BBB (Fig. 1). The integral components of the tight junction are claudins-1, -3, -5 and -12 and occludin which are directly responsible for determining permeability of the tight junction. Therefore, the degree of tightness is determined by interactions of tight junction family members on endothelial cells.

## Expression of claudin-5

Claudin-5 is localised specifically to the endothelial cell layer in the brain, lung [9], liver [10], kidney [11] and skin [12] while it is expressed in the epithelial cell layer in the stomach (Table 1). In peripheral tissues, claudin-5 displays greater heterogeneity in tissue expression; claudin-5 is expressed in enterocytes in the small and large intestine and in pancreatic acinar cells [10]. During embryonic development claudin-5 is transiently expressed in the retinal pigment epithelium (RPE) where its expression correlates with permeability changes in the developing RPE [13]. Claudin-5 expression was also shown in CNS lymphatic vessels where it assumed a more diffusive, punctate staining pattern like diaphragm lymphatic vessels; while contrasting with the continuous expression pattern in meningeal blood vessels [14, 15]. Claudin-5 is expressed in many tissues with the highest expression observed in the brain and lungs [16]. The embryonic development of claudin-5 expression has been described in chicks. Within chicks, only claudin-5 is localised to endothelial cells and is present from the onset of vasculogenesis at HH stage 4 in extraembryonic tissue. Claudin-5 expression appears in the embryo at HH stage 8 coinciding with embryonic vascularisation and by HH stage 24 is highly visible in branching blood vessels in the brain [17]. In the developing rat, claudin-5 is detected as early as E12 coinciding with angiogenesis [18]. In humans, during foetal development, claudin-5 is initially detected diffusely in the cytoplasm of endothelial cells. By 14 weeks of development, claudin-5 shifts from the cytoplasm to discontinuously label the interface of neighbouring endothelial cells with more continuous staining patterns evident by 18 weeks gestation [19]. Claudin-5 expression continues to increase during post-natal development. Signalling molecules secreted from endothelial cells, pericytes and astrocytes during barriergenesis and BBB maturation from E10 to postnatal establish and maintain claudin-5 levels in the CNS. Of these, hedgehog signalling plays a key role in maintenance of barrier properties. Astrocytes are the primary source of sonic hedgehog (SHH) and SHH, secreted from astrocytes, binds to endothelial patched homolog 1 receptor, releasing the inhibitory effect of smoothened on Gli transcription and culminating in expression of SHH-regulated genes including claudin-5, VE-cadherin and occludin [20]. Pericyte-endothelial crosstalk mediated by platelet-derived growth factor β and transforming growth factor β (TGF-β) signalling leads to maintenance of BBB integrity [21–23]. CD146 has been identified as a key spatiotemporal molecule orchestrating BBB development. Initially, CD146 is expressed in brain endothelial cells of developing vessels where it is a critical regulator of claudin-5 expression and BBB permeability. Subsequently, CD146 expressed in pericytes promotes pericyte coverage of endothelial cells and BBB integrity by directly regulating PDGFβ/PDGFRβ signalling. In addition, silencing of CD146 downregulates claudin-5 expression in brain endothelial cells suggesting that CD146 is necessary for claudin-5 expression [24]. Maintenance of tight junction integrity is achieved through bidirectional signalling between pericyte-endothelial cells and astrocyte-endothelial cells into adulthood [25]. While claudin-5 is expressed in all CNS vessels, there is considerable heterogeneity of expression within each vessel with maximal expression observed in capillaries and small venules and minimal expression observed in larger venules [26]. Within brain capillary endothelial cells, claudin-1, 3 and 12 mRNAs have been detected in addition to claudin-5. However, mRNA levels of claudin-5 are approximately 600 times higher compared to claudin-1, -3 and -12 [16, 27]. While a 3D structure of a brain associated claudin protein has yet to be published, recently the crystal structure of claudin-15 has been determined which is in the same class of claudin as claudin-5. This model reveals that claudin-5 may contain a β-sheet consisting of the two extracellular domains tethered to the transmembranous 4 helix bundle [28].

**Table 1:** Tissue distribution and function of claudin-5

Tissue	Function	Pathology	Observations
Endothelial tissue e.g. brain capillary, lung endothelium	BBB—decrease paracellular permeability	Downregulated in schizophrenia, glioblastoma, Alzheimer’s disease Hemizygous in 22q11DS	Mouse KO—post natal lethality, increased permeability < 800 Da [29] Mouse KD—seizure, depression, inflammation [30, 31]
Peripheral lymph node	Decrease permeability	–	Claudin-5 ± mice – increased leakage and inflammation following UV exposure [32]
Cardiomyocytes	Unknown	Downregulated in heart failure [33]	Dystrophin KO – overexpression of claudin-5 prevents cardiomyopathy [34]
Pancreatic ductal and acinar cells	Unknown	–	–
Retinal pigment epithelium	Changes in permeability in developing RPE	–	–
Small/large intestine	Unknown	–	–
Stomach epithelium	Unknown	–	–
Seminiferous epithelium	Blood-testis barrier	–	Claudin-5 downregulation and biotin tracer leakage in Etv5 KO mice [35]
Ovarian epithelium	Theca vascular development	Overexpression associated with aggressive, high-grade ovarian adenocarcinoma [36]	–
Prostate	Unknown	Overexpressed in prostate adenocarcinoma [37]	–

## Structure and interactions of claudin-5

**Fig. 2:**
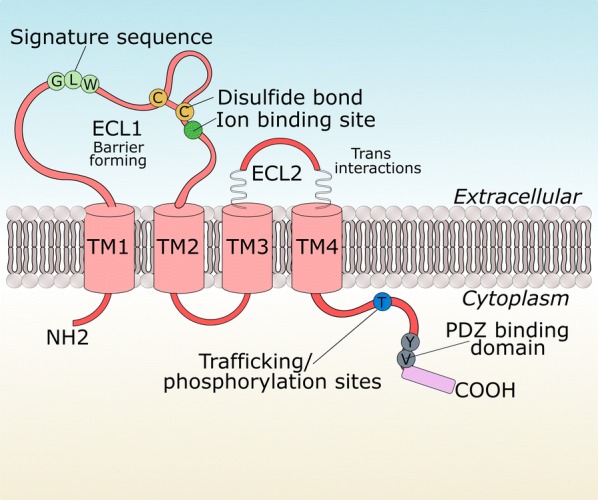
Structure of claudin-5. Claudin-5 consists of 4 transmembrane domains (TM), a short NH_2_ terminus, two extracellular loops (ECL), a short intracellular loop and a longer COOH terminus. ECL1 contains a disulphide bond and ion binding site as well as a highly conserved signature motif. The long COOH terminus contains the PDZ binding motif for interactions with scaffolding proteins including ZO-1, -2 and -3. Additionally, the COOH terminus contains trafficking and phosphorylation residues (Adapted from [44])

The *CLDN5* gene is located on chromosome 22 in humans and chromosome 16 in mice. Claudin-5 is a member of the claudin multigene family of which there are up to 27 members. Each family member can be subdivided into two groups based on sequence similarity and proposed function. Group one contains the classic claudins (1–10, 14, 15, 17, 19) and group two contains the non-classic claudins (11–13, 16, 18, 20–24). Claudins are similar in structure to the tight junction proteins occludin, tricellulin, and connexins as they contain four transmembrane domains, despite sharing minimal sequence homology. The protein product of the gene is a 23 kDa integral membrane protein that consists of four transmembrane domains, two extracellular loops (ECL) an intracellular NH_2_ terminus, a long COOH terminus and a short intracellular loop (Fig. 2). The first extracellular loop (ECL1) of claudins is known to be vital to the barrier sealing properties of tight junctions. Mutations to conserved cysteine residues in ECL1 of claudin-5 in MDCK cells results in increased paracellular permeability to mannitol and monosaccharides [38]. The second extracellular loop (ECL2) has been less intensively studied however, for claudin-5, it has been proposed to be involved in strand formation via trans interactions [39]. The amino acid residue Y158 was found to be necessary for homophilic trans-interaction while mutations to other residues was found to reduce contact enrichment (I142T, D149 N/T151L) [40]. Mutations of ECL2 residues and subsequent transfection into HEK cells disrupted the enrichment of claudin-5 at cell contacts with other claudin-5-expressing cells. A recent study has pinpointed a role of ECL2 in mediating turnover of claudin-5 protein [41]. Claudins associate with claudin species on adjacent cells as well as forming cis interactions on the same cell [42, 43]. Claudins are a major structural component of the tight junction resulting from homotypic to heterotypic interactions via their extracellular domains [44, 45]. Adhesion of endothelial cells to the extracellular matrix (ECM) is mediated, in part, by interaction of the adhesion receptor β1-integrin on endothelial cells to ECM proteins such as laminin, collagen IV and perlecan. Blockade of this interaction leads to time-dependent decreases in claudin-5 expression in isolated mouse brain microvascular endothelial cells [46]. The spatial organisation of claudin strands is determined by the zonula occludens (ZO) scaffolding proteins with most claudin species containing a C terminus PDZ-binding motif which can bind to PDZ motifs on the ZO proteins [47] linking them to the actin cytoskeleton. In the human endothelial cell line hCMEC/d3, claudin-5 was found to interact with caveolin-1, VE-cadherin, p120 catenin and Gai2, although surprisingly it did not associate with ZO proteins, claudin-3 or MUPP-1 [48]. Trafficking of claudin-5 to the apical membrane is dependent on the C terminus as truncated proteins lacking the entire C terminal tail are retained intracellularly in the endoplasmic reticulum. Interestingly, deletion of just the PDZ motif from the C terminal tail still results in localisation to the apical membrane, suggesting that the 15 juxtamembrane cytosolic residues (amino acids 182–196) are involved in mediating tight junction localisation [49]. Folding and assembly of claudin-5 protein into the tight junction is controlled by non-conserved residues in the third transmembrane and ECL2 segments [40]. The C terminus of claudin-5 has also been shown to be bound by Clostridium perfringens endotoxin resulting in internalisation of claudin-5 to the cytoplasm and reduction of transendothelial electrical resistance (TEER) [50].

## Function of claudin-5

The paracellular sealing function of claudin-5 stems from its association with claudin proteins on neighbouring endothelial cells. Tight junctions provide one mechanism of cell to cell adhesion through their shared interactions with other tight junction strands on opposing cell membranes at so called “kissing points” to reduce the intercellular distance to almost zero. In essence, tight junctions form a mechanical link between individual endothelial cells to maintain the structural integrity of the vasculature and to prevent the diffusion of solutes and ions through the intercellular space. The result of this tight construction is a high electrical resistance of ~ 1500–2000 Ω·cm^2^ [51, 52]. During embryonic development between E11 and E13, there is a dramatic change in permeability in the CNS. Fenestrations, which are characteristic of leaky blood vessels, disappear and are replaced by tight junctions [53, 54]. Located apically towards the vessel lumen, tight junctions form homo- and heterotypic interactions with tight junctions on adjacent endothelial cells. Claudin-5 plays a key role in the paracellular barrier to small molecules. Transfection of claudin-5 into Caco-2 cells contributed to barrier function [55]. Exogenous expression of claudin-5 in cultured rat brain endothelial cells reduced the permeability to inulin [56]. The generation of animal knockouts has revealed the size-selective properties of claudin-5. Knockout of claudin-5 in mice allowed rapid extravasation of tracers with molecular weights < 800 Da from the intravascular compartment. Importantly, the barrier remained impermeable to microperoxidase (1.9 kDa). All claudin-5 knockout mice die within 10 h of birth. Despite this, the tight junction remains intact in knockout mice [29]. Exposure of claudin-5 heterozygous knockout mice to low dose ultraviolet radiation revealed exacerbated skin inflammation and lymphatic vessel leakage [32]. Double knockdown of claudin-5 with occludin facilitates enhanced permeation of tracer molecules between 3 and 10 kDa [57].

## Regulation of claudin-5

Claudin-5 expression is regulated by several upstream signalling pathways at the transcriptional and post-translational levels (Table 2). Additionally, regulation of claudin-5 and of tight junction properties in general occurs via physical interactions with cytoplasmic scaffolding proteins and interactions with proteins on the same (cis) or adjacent cells (trans). Together, these processes determine claudin-5 assembly, remodelling and degradation.

**Table 2:** Regulators of claudin-5 expression

Regulators	Effect on expression	Mechanism
Phosphorylation	Decrease	Thr207 PKA/PKC signalling
VEGF	Decrease	Basolateral VEGFR2-induced downregulation via p38
TGF-β	Decrease	ALK signalling/MMP upregulation
GDNF	Increase	–
Adrenomedullin	Increase	–
Glucocorticoid	Increase promoter activity	Glucocorticoid response elements in claudin-5 promoter
SOX-18	Increase promoter activity	SOX binding in claudin-5 promoter transactivate claudin-5
TNFα	Decrease	NFκB dependent repression
Estrogen	Increase	Estrogen receptor/SP1 binding in claudin-5 promoter
MMP	Degradation	–
Fox01	Repress transcription	Nuclear accumulation and repression of claudin-5 transcription
cAMP	Increase cell–cell localisation	PKA-dependent and independent pathways
SHH	Increase	SHH binding to patched releases inhibitory effect of Gli on downstream targets including claudin-5
VE-cadherin	Increase	Prevent Fox01/β-catenin mediated repression
Ubiquitination	Degradation	Ubiquitination at L1999. Proteosome-mediated degradation
CCL2	Turnover	Caveolin-dependent removal
Glutamate	Decrease	NMDAR-mediated-cPLA_2_ activation and MMP upregulation
Autophagosome	Turnover	ECL2-dependent endocytosis

## Phosphorylation

Phosphorylation events are important in regulating claudin-5 protein expression. Several phosphorylation sites have been identified on the claudin-5 protein sequence. RhoK phosphorylates claudin-5 cytoplasmic domains at T207 leading to functional impairment of the BBB in cultured endothelial cells [58]. Forkhead box protein 01 (Fox01) is a negative regulator of claudin-5 expression. Acting in concert with β-catenin; nuclear accumulation represses claudin-5 expression. Fox01 and β-catenin bind to claudin-5 promoter regions to inactivate transcription. This process is also dependent on VE-cadherin, a positive regulator of claudin-5 expression. VE-cadherin-mediated AKT activation results in phosphorylation of Fox01 and its exclusion from the nucleus [59]. Further interrogation of this signalling pathway revealed VE-cadherin inhibits polycomb repressive complex-2 (PRC2) protein activity on the claudin-5 gene promoter and that Fox01 acts to stabilise polycomb protein binding to the claudin-5 promoter. Additionally, VE-cadherin sequesters Ezh2, a core subunit of PRC2, at the cell membrane, preventing its nuclear translocation. Inhibition of VE-cadherin sequestration of Ezh2 lead to downregulation of claudin-5 [60]. IL4 exerts a similar effect via activation of Jak/STAT6 signalling and subsequent phosphorylation of Fox01 leading to upregulation of claudin-5 [61]. Protein kinase A (PKA) signalling is a well characterised pathway regulating BBB integrity and claudin-5 levels. In brain endothelia, cyclic AMP (cAMP) enhances claudin-5 gene expression and promotes the protein presence at cell–cell contacts via PKA dependent and independent pathways resulting from phosphorylation of claudin-5 at Thr207 on the intracellular carboxyl terminal. Despite claudin-5 enhancement at cell borders upon cAMP treatment, barrier properties were diminished with reductions in TEER and increased permeability to mannitol [62, 63]. Modulation of claudin-5 expression and paracellular permeability are also affected by PI3K signalling. Mouse brain endothelial cells exposed to 3-chloropropanediol show increased expression of PI3K resulting in phosphorylation of AKT and culminating in loss of membrane association of claudin-5 to a more diffuse cytosolic pattern [64]. Furthermore, this effect was attenuated with the use of selective PI3K inhibitors. Another signalling pathway that mediates BBB impairment through claudin-5 regulation is Rho/ROCK due to phosphorylation of tyrosine residues. This process appears to be a mediator of monocyte infiltration of the CNS during HIV-1 encephalitis [65]. CCL2 or monocyte chemoattractant protein-1 regulates claudin-5 expression in cultured endothelial cells via Ser/Thr phosphorylation, a process dependent on interaction between protein kinase C alpha and Rho [66]. PKC signalling also regulates tight junction expression patterns in normoxia and hypoxic conditions. Under hypoxic conditions, loss of junctional integrity occurs via PKC signalling [67]. Phosphorylation of NFκB leads to upregulation of matrix metalloproteinase 9 (MMP9) via NFκB binding to MMP9 promoter regions and subsequent degradation of claudin-5 [68]. MMPs are acutely involved in the degradation of junctional components as will be discussed below.

## Growth factors

Vascular endothelial growth factor (VEGF) is a potent regulator of claudin-5 expression. VEGF-A treatment of brain microvascular endothelial cells induces downregulation of claudin-5. Local injections of VEGF-A to brain parenchyma disrupts claudin-5 expression along the vasculature and leads to extravasation of serum components [69]. VEGF-A-induced barrier dysfunction was also found to be dependent on the polarity of brain endothelial cells with parenchymal VEGF-A inducing leakage with no effect following intravenous injection in mice. Furthermore, in polarised brain microvascular endothelial cells grown on transwell filters; basolateral but not apical administration of VEGF-A induced redistribution of claudin-5 in cellular monolayers [70].

TGF-β also has a crucial role in vascular development and homeostasis. TGF-β signalling pathways regulate microvascular homeostasis through two distinct receptors; activin receptor-like kinase (ALK)-1 and ALK5. Indeed, ALK1 and ALK5 have opposing effects on microvascular stability with the ALK1 pathway promoting cellular proliferation and permeability while ALK5 inhibits permeability [71]. Activation of ALK5 signalling by TGF-β leads to downregulation of claudin-5 through SMAD2/3. Inhibition of ALK signalling with the ALK5 specific inhibitor SB-431542 rescues claudin-5 expression in embryonic endothelial stem cells [72, 73]. In the context of inflammatory pain, pharmacological inhibition of ALK5 increased the expression of claudin-5 while reducing nuclear accumulation of SMAD2/3 [74]. TGF-β mediated downregulation of claudin-5 may also result from activation of MMP9; as brain endothelial cells treated with TGF-β show MMP9 upregulation, concomitant downregulation of claudin-5 and increased permeation of FITC-dextran. Treatment with SMAD3 inhibitors attenuated this effect, reinforcing the role of ALK signalling in regulation of claudin-5 levels [75]. TGF-β has also been shown to directly modulate claudin-5 levels via phosphorylation of tyrosine residues resulting in increased permeability in human endothelial cells [76].

Other growth factor molecules have also been shown to regulate claudin-5 levels and barrier properties. Glial cell line-derived neurotrophic factor, a member of the TGF-β superfamily, secreted from pericytes, increased expression of claudin-5 in an in vitro human BBB model and enhanced barrier properties [77]. Adrenomedullin, a potent vasodilator and hypotensive peptide, dose dependently increases expression of claudin-5 in isolated rat brain endothelial cells while increasing TEER and decreasing flux of sodium fluorescein (376 Da) and Evans’ blue (960 Da) [78].

## Transcriptional regulation

Cloning and characterisation of the murine claudin-5 promoter has identified numerous regulatory motifs including glucocorticoid response elements and NFκB binding sites that potentially regulate claudin-5 expression [79, 80]. Interestingly, E-box sequences were found in the promoter region, suggesting a role for circadian transcription factors in regulating claudin-5 levels [79]. Indeed, Per2 (a transcriptional repressor of circadian rhythms) mutant mice have increased blood-retina barrier permeability and redistribution of claudin-5 in the retinal vasculature [81]. Additionally, claudin-5 displays rhythmic expression throughout the day in normal mice but this was absent in mice with endothelial specific deletion of the clock transcription factor Bmal1 (a transcriptional activator of circadian rhythms) [82]. These studies suggest that claudin-5 may be a circadian clock-controlled gene.

TNF alpha induces vascular permeability through induction of NFκB signalling culminating in repression of mouse claudin-5 promoter activity [83]. Treatment of bovine retinal endothelial cells with IL-1β or TNFα induced permeability, downregulation and altered localisation of claudin-5 and ZO-1. Chemical or viral-mediated blockade of NFκB signalling attenuated junctional remodelling and reduced permeability [84].

The claudin-5 promoter also contains estrogen response elements that can be bound by the classical estrogen receptor (ER) subtypes ERα and ERβ to induce claudin-5 expression [85, 86]. Treatment of murine brain endothelial cells with 17 beta-estradiol increases claudin-5 promoter activity and protein expression. Administration of estrogen increases the expression of claudin-5 and occludin in ovariectomised rodents, while ERβ knockout mice have reduced levels of claudin-5 [87]. In the setting of experimental ischemic stroke, activation of G protein-coupled estrogen receptor 1 ameliorated BBB permeability following four vessel occlusion in ovariectomised rats and restored expression levels of claudin-5 [88]. Similarly, tight junction proteins are differentially regulated by ER subtype-specific agonists in the setting of experimental stroke. Activation of ERα suppresses the reduction of occludin while activation of ERβ attenuates reductions in claudin-5 [89].

The gut microbiota is also a critical regulator of claudin-5 expression and BBB function and integrity. Lack of a normal gut microbiota in germ-free mice is associated with increased BBB permeability and decreased expression of tight junction proteins including claudin-5. Moreover, this phenotype is observed from development into adulthood suggesting that crosstalk between the brain and gut microbiota necessitates normal BBB development. Introduction of flora from pathogen-free mice with a gut microbiota to germ free mice was associated with decreased BBB permeability and increased expression of claudin-5 and occludin [90].

## Turnover

Turnover of claudin-5 protein is continuous with the protein having a relatively short in vitro half-life of 90 min, yet the details of remodelling are unclear [91]. During stroke, claudin-5 is removed from the endothelium in a caveolin-dependent process [92] as well as in cultured endothelial cells treated with the cytokine CC-chemokine ligand 2 (CCL2) before further processing in early but not late endosomes [93]. Ubiquitination at L199 targets claudin-5 for degradation via proteasomal pathways [91]. How tight junction remodelling occurs without loss of barrier integrity has remained elusive. A recent study may provide clues and implicates autophagy-mediated degradation of claudin-5 in the process. The presence of claudin-5 in large double membraned vesicles was confirmed in MDCK cells transfected with claudin-5 tagged with YFP and TRQ. Crossover endocytosis occurs where claudin-5 particles from adjacent endothelial cells are endocytosed into the opposing cell and trafficked to the autophagolysosome for subsequent degradation. This process was dependent on claudin–claudin interactions with amino acid substitutions in the 2nd extracellular loop impairing the process [41].

In the oxygen–glucose deprivation model of ischaemia–reperfusion, caveolin-1 mediates claudin-5 dissociation from the plasma membrane and barrier dysfunction. Inhibiting autophagy with bafilomycin A1 or 3-methyladenine attenuated claudin-5 degradation, while inhibiting nitric oxide prevented caveolin-1 translocation and claudin-5 degradation. This model further supports a role for autophagic degradation of claudin-5 in normal homeostasis and pathology [94].

Treatment of brain endothelial cells with the GSK3β inhibitor lithium chloride increases barrier properties concomitant with an increase in the amount of claudin-5 and occludin. This process was attributed to reduced protein turnover owing to increased half-life of claudin-5 and occludin [95].

## Dynamic tight junction remodelling in disease

### Neuroinflammation

**Fig. 3:**
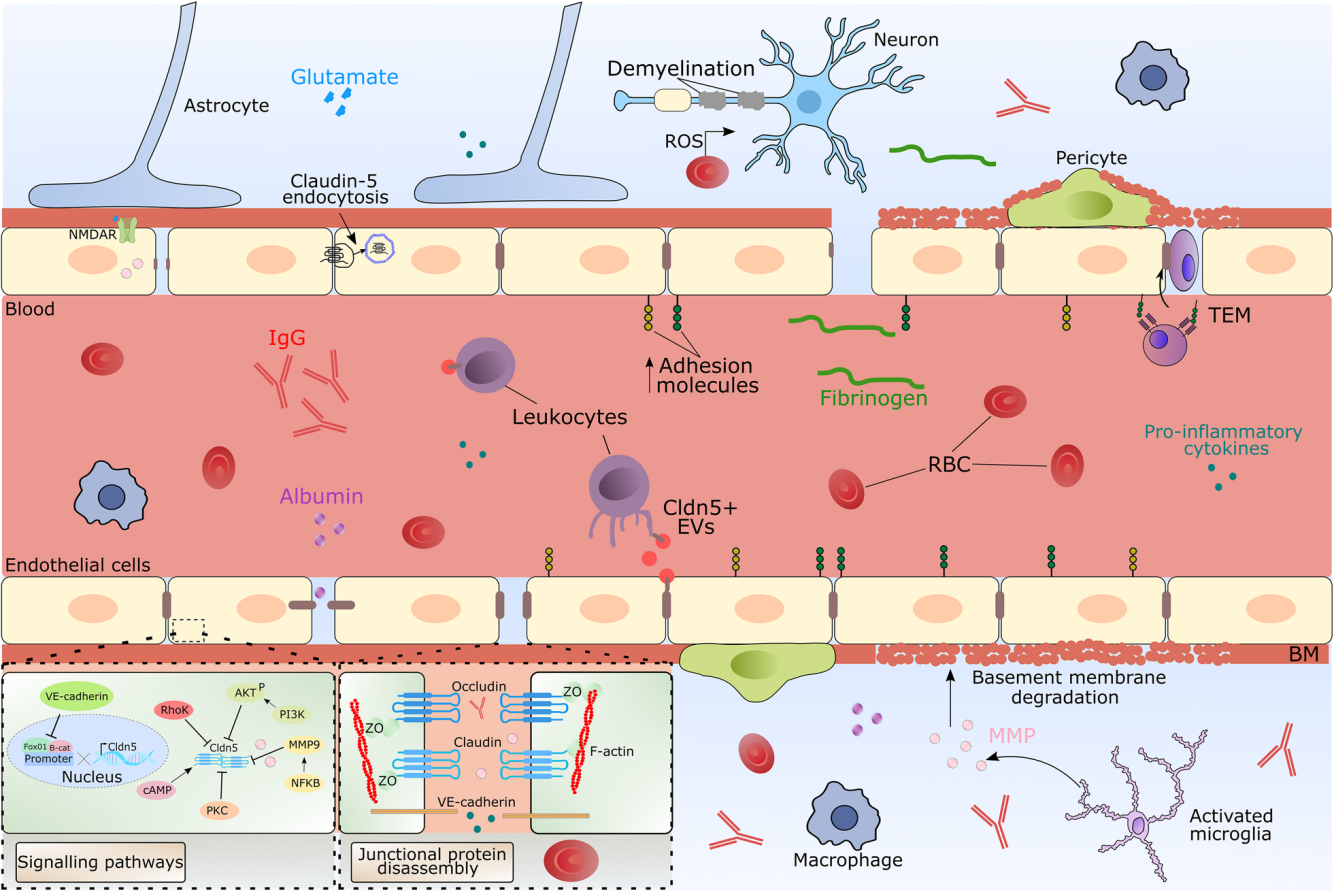
Dynamic tight junction remodelling in disease. Breakdown of the blood–brain barrier (BBB) and loss and mis-localisation of tight junction proteins leads to immune cell entry to the central nervous system (CNS). In multiple sclerosis, this results in neuroinflammation, neurodegeneration and disease progression and transendothelial migration (TEM) of peripheral blood leukocytes. Claudin-5 positive extracellular vesicles (EV) can bind to blood leukocytes to potentially facilitate TEM of leukocytes into the CNS. BBB breakdown also leads to the perivascular accumulation of plasma-derived proteins such as fibrinogen, albumin and immunoglobulin G (IgG) that is found in humans with temporal lobe epilepsy as well as in rodents injected with the seizure-inducing agent kainic acid. In rodents, glutamate released from neurons and astrocytes can bind to* N*-Methyl-d-aspartate receptors (NMDAR) on the brain endothelium and regulate tight junction proteins claudin-5 and occludin via upregulation of matrix metalloproteinases (MMP). Extravasation of red blood cells (RBC) following traumatic brain injury releases toxic haemoglobin and free iron culminating in generation of reactive oxygen species (ROS). Extravasation of fibrinogen and albumin activates microglia leading to secretion of MMP and basement membrane (BM) degeneration. Dashed boxes display the signalling pathways and molecules that regulate expression of claudin-5 and subsequent disassembly of the tight junction protein complexes that facilitates paracellular BBB permeability of blood-derived molecules

Loss of BBB integrity is an early and prominent pathological feature of neuroinflammatory disorders (Fig. 3). Loss of BBB integrity can lead to dysfunction of fluid homeostasis, oedema and facilitates the CNS entry of inflammatory mediators, antibodies, plasma and serum proteins and anaphylatoxins culminating in neuroinflammation [96]. BBB permeability is increased in response to many proinflammatory stimuli such as lipopolysaccharide, tumor necrosis factor α (TNFα), IL-6, MCP-1 and IL-1β, with concomitant downregulation of tight junction proteins [97, 98]. In experimental autoimmune encephalomyelitis (EAE), loss of claudin-5 is a central event in determining BBB disruption owing to overexpression of VEGF-A from glial fibrillary acid protein positive astrocytes [69]. Microglia are the resident immune cell of the CNS and constantly survey the brain parenchyma by sampling their microenvironment with highly motile processes [99]. However, in response to danger signals, brain endothelial cells become activated and are characterised by upregulated expression of cell adhesion molecules such as ICAM-1 and VCAM-1 to facilitate leukocyte entry to the CNS and facilitate an immune response. Transendothelial migration (TEM) of peripheral leukocytes then progresses through paracellular channels via reorganisation of junctional complexes and the actin cytoskeleton. Rapid remodelling of the tight junction occurs within 10 min of TEM with discontinuous claudin-5 staining in areas of monocyte TEM [100].

In an elegant study using high-resolution 3D confocal microscopy, Paul et al. identified a novel mechanism of leukocyte TEM potentially mediated by claudin-5 protein bridges during EAE. Leukocytes bearing claudin-5 were found in the blood and spinal cord of mice with EAE. Using eGFP:claudin-5 transgenic mice, the endothelial origin of claudin-5 positive leukocytes was determined [101]. Furthermore, extracellular vesicles (EV) from mice with EAE as well as brain endothelial cells treated with TNFα contained claudin-5 and could bind to leukocytes in vitro. These results suggest that during neuroinflammation; EVs could act as a vehicle for endothelial to leukocyte transfer of claudin-5 protein at the BBB and facilitate TEM into the CNS [101]. In support of this novel role for claudin-5, peripheral blood leukocytes isolated from patients with multiple sclerosis as well as healthy controls expressed claudin-5 with elevated levels in patients experiencing disease relapse. Additionally, anti-inflammatory treatment with glucocorticoids was accompanied by decreased leukocyte expression of claudin-5 [102]. This suggests a correlation between disease severity and claudin-5 protein expression in circulating leukocytes. These studies highlight the active role of the BBB in mediating contact between the CNS and peripheral immune system and point to a more multifaceted role for claudin-5 in regulating CNS homeostasis.

The paracellular pathway of the BBB can also be hijacked by viruses and pathogens to infiltrate the CNS. HIV-1 infection is associated with disrupted claudin-5 and occludin integrity which correlate with monocyte infiltration of the CNS [65]. In humanised mice, HIV-1 infection is associated with impaired SHH signalling, downregulated expression of claudin-5 and leakage of Evans’ blue dye while an agonist to smoothened rescues this loss [103]. Similarly, West Nile Virus (WNV), a mosquito-borne flavivirus that causes neuroinflammation, neurodegeneration and death, induces loss of tight junction proteins and upregulation of MMPs in WNV-infected mice [104]. Treatment of cultured brain endothelial cells with interferon λ stabilises claudin-5 and ZO-1 localisation after WNV infection while treatment of WNV-infected mice with interferon λ restricts CNS invasion of WNV [97]. Group B streptococcus is the leading cause of neonatal meningitis and disrupts the paracellular pathway of the BBB by downregulating claudin-5, occludin and ZO-1 via upregulation of Snail1. Snail1-dependent decreases in tight junctions facilitated bacterial passage through disrupted tight junction complexes [105].

### Multiple sclerosis

Multiple sclerosis (MS) is an autoimmune disorder where immune cells including leukocytes, T cells, B cells and peripheral macrophages attack the myelin sheath surrounding axons by entering across a damaged BBB [26]. Transmigration of immune cells across the BBB is an early and prominent feature of MS models. *Post*-*mortem* studies have revealed that BBB dysfunction is an early feature of MS; confirmed by loss of tight junction proteins (claudin-5, occludin and ZO-1) in different stages of MS. Loss of tight junctions was associated with fibrinogen leakage [106, 107]. In fact, studies from human MS plaques and animal models have revealed that BBB integrity is disturbed as an early event preceding the influx of pathogenic T cells and correlates with clinical severity of EAE [108]. In vitro, treatment of mouse brain endothelial cells with serum isolated from MS patients caused downregulation of claudin-5 and occludin, upregulation of MMP9 and increased permeability; this effect could be partially rescued by glucocorticoid treatment [109].

EAE is an animal model of CNS inflammation commonly used to investigate the neurobiology and pathogenesis of MS. Loss of claudin-5 and BBB integrity occurs during early and late disease pathology with an associated reduction in total claudin-5 protein. Transmigration of leukocytes occurs primarily in the post-capillary venules and is associated with the loss of claudin-5 [26]. Moreover, by using eGFP-claudin-5^+/−^ transgenic mice that express a fusion of eGFP to claudin-5 in endothelial cells, in conjunction with intravital two-photon microscopy; it was possible to visualise the dynamic remodelling of the tight junction at the blood-spinal cord barrier during EAE. Loss of claudin-5, characterised by increased protrusions, gaps and tortuous segments, was apparent before EAE onset and persisted throughout disease. Additionally, loss of claudin-5 was associated with transmigration of Th17 lymphocytes across the BBB and into the CNS [110]. In a rat model of EAE, administration of an antagonist of purinergic P2X receptors attenuated clinical hallmarks of the disease and rescued claudin-5 expression [111]. Expression of interferon γ appears to be protective during EAE by increasing expression of occludin and ZO-1 and increasing membrane localisation of claudin-5. However, silencing claudin-5 expression abolished interferon γ-mediated protection [112]. Restoration of junctional components, in particular claudin-5, in EAE appears to be a promising approach to preventing disease progression by attenuating TEM of immune cells. Indeed, treatment with orally available LY-317615, a small molecule inhibitor of protein kinase Cβ, ameliorates inflammation, demyelination and axonal damage through induction of claudin-1, -3, -5 and ZO-1 [113]. Treatment of mouse brain endothelial cells with LY-317615 induced the most striking changes in claudin-5 mRNA with a fivefold upregulation. In vivo, two-photon imaging revealed a reduction in FITC-dextran permeability and increased vascular claudin-5 staining suggesting that this small molecule therapeutic attenuates TEM via upregulation of tight junction protein claudin-5.

### Stroke and traumatic brain injury

Rodent models have revealed that stroke and traumatic brain injury (TBI) are characterised by a biphasic opening of the BBB with downregulation of tight junction proteins including claudin-5, occludin and ZO-1 and subsequent extravasation of serum proteins [114]. Mimicking hypoxic conditions of stroke in vitro revealed downregulation of claudin-5 and altered localisation accompanied by decreased TEER; while hypoxic conditions in the retina downregulated claudin-5 and increased the extravasation of low molecular weight tracers [115]. Elevated levels of MMPs results in fragmentation and re-localisation of claudin-5 and occludin during transient focal ischaemia following 3 h reperfusion; an effect blocked by inhibition of MMP [116]. By using sophisticated tracer experiments coupled with two-photon time-lapse imaging in the transient middle cerebral artery occlusion model, the dynamic events at the BBB leading to barrier deficits after stroke have been mapped. Using eGFP:claudin-5 transgenic mice to selectively visualise endothelial tight junctions, ultrastructural changes including gaps between adjacent endothelial cells and absence of tight junctions was apparent 48–58 h after stroke but not before [117]. Moreover, these changes correlated with the delayed leakage of biocytin-TMR and IgG. Earlier timepoints were however, associated with increased rates of endocytosis and transcytosis as early as 6 h after insult with minimal deficits in tight junction integrity [117]. This dynamic sequence of events at the BBB highlights the involvement of transcellular and paracellular pathways in modulating BBB permeability in the hours and days after stroke. Similar findings are apparent in the penumbral region surrounding the site of TBI. In a cortical cold-induced model of TBI, enhanced caveolin-1 expression preceded downregulation of tight junctions claudin-5 and occludin [118]. Following siRNA mediated downregulation of claudin-5 in the cold-induced model of TBI, reduced claudin-5 expression enhances the movement of water from brain to blood, reducing cerebral oedema and accelerating neurological recovery [119].

The impact of repeated head trauma in sport and military veterans is also under increasing scrutiny. In chronic traumatic encephalopathy, a condition arising from repetitive mild TBIs, deposition of hyperphosphorylated tau protein is associated with downregulation of claudin-5 and extensive BBB dysfunction [120, 121].

### Epilepsy

Epilepsy is a common neurological disorder characterised by recurrent, unprovoked, spontaneous seizures. Many pathological processes are involved in the generation of spontaneous seizures, including neuroinflammation and cell loss [122]. BBB dysfunction is a hallmark pathology of many neurological conditions. Moreover, the degree of BBB dysfunction often correlates with disease severity. This is particularly true in epilepsy, where BBB dysfunction and microvascular permeability of serum macromolecules is one of the earliest events following status epilepticus [123] and the degree of BBB dysfunction correlates with seizure severity [124]. In fact, recent studies provide evidence to the hypothesis that a leaky barrier contributes to the generation of seizures, leading to more seizures which trigger further barrier dysfunction leading to epilepsy progression [124–126]. There is little understanding of the role of the tight junction in epileptogenesis or following seizure events with inconsistent findings between animal models and human samples. Following cerebroventricular injection of kainic acid (a glutamate receptor agonist that induces seizures), increased levels of claudin-5 and ZO-1 were observed in the hippocampus. This may result from the increased density of blood vessels associated with this model [127]. Another study found reduced levels of claudin-5 within 12 h of kainic acid injection with increased levels observed after 3 days. However, by 3 days, claudin-5 in the vasculature remained diffuse and mis-localised indicating disrupted junctional integrity [128]. These findings may implicate an immediate loss of BBB integrity due to downregulation of claudin-5 as a precipitating factor in the subsequent disease progression of the model. In surgically resected brain tissue from individuals with refractory drug resistant epilepsy, claudin-5 staining was diffuse and discontinuous in blood vessels [129]. Angiogenesis is a prominent feature of human temporal lobe epilepsy and dysfunction of claudin-5 in these immature and leaky vessels may facilitate immune cell infiltration and neuroinflammation associated with the condition [130]. In the pilocarpine model of epilepsy, downregulation of tight junctions claudin-1, -5, occludin and ZO-1 was accompanied by upregulation of MMP-2 and -9 while ex vivo application of glutamate to isolated capillaries was sufficient to induce tight junction downregulation and increased BBB permeability [131].

### Psychiatric disorders

There is abundant evidence implicating BBB dysfunction and vascular abnormalities in psychiatric disorders [132]. Claudin-5 is also known as transmembrane protein deleted in velocardiofacial syndrome. It was originally identified as a gene deleted on chromosome 22q11.21 in a region deleted in the chromosomal abnormality 22q11.21 deletion syndrome (22q11DS), previously known as velocardiofacial syndrome [9]. 22q11DS is notable for the 30-fold increased risk of schizophrenia compared to the general population. Several studies have pinpointed a single nucleotide polymorphism (SNP) in the 3′ untranslated region of the claudin-5 gene that is linked to schizophrenia [133–135]. Rs10314 is a G to C base change and analysis of the effect of this SNP on claudin-5 protein levels revealed reduced levels of the C SNP following transfection into HEK cells [30]. In post-mortem specimens from individuals with schizophrenia, claudin-5 protein levels were decreased in the frontal cortex and this reduction was associated with PKA signalling [136]. Numerous research groups have pinpointed a synergistic relationship between BBB disruption and immune cell entry to the brain mediating neuroinflammatory insults and psychiatric disorders such as depression [137]. Claudin-5 has emerged as a potential mediator of these effects with several studies identifying downregulation of claudin-5 in numerous models of depression [138, 139]. Transient knockdown of claudin-5 in the nucleus accumbens is associated with exacerbated depression-like behaviours in the chronic social defeat model of depression [31]. Downregulation of claudin-5 protein coupled with stress-induced recruitment of peripheral immune signals resulted in increased BBB permeability and passage of blood proteins such as IL6 and the development of depression-like behaviours. Additionally, these effects are potentiated by drugs of abuse such as methamphetamine [140].

### Pain disorders

Recent studies have highlighted the functional alterations of the BBB in relation to pain disorders, including inflammatory pain and migraine. It is hypothesised that peripheral tissue damage resulting in pain releases numerous factors that can induce BBB permeability such as IL-1β, TNF-α and histamine. Several groups have shown that acute and chronic inflammatory pain significantly alters BBB permeability and causes remodelling of tight junction components. Rats injected with formalin, γ-carrageenan or complete Freund’s adjuvant (CFA) to mimic acute, short-term and chronic inflammatory pain displayed elevated [^C^14] sucrose BBB permeability. Moreover, peripheral inflammation enhanced ZO-1 expression in all models while occludin was downregulated in short-term and chronic models [141]. Others have reported similar findings along with large increases in claudin-3 and -5 expression following chronic inflammatory pain [142]. Tight junction remodelling appears to be largely time-dependent as claudin-5 is downregulated 24 h following CFA and upregulated at 48–72 h following inflammatory pain induction. However at these timepoints claudin-5 is no longer localised to the junction and displays a more diffuse pattern of expression [143]. These effects could be abolished by nociceptive inhibition with the local anaesthetic bupivacaine [144]. Local injection of γ-carrageenan to the rat hind paw to induce peripheral inflammatory pain is associated with increased BBB permeability and increased protein expression of claudin-5 and ZO-1 and decreased levels of occludin as shown before. Pre-treatment of rats with recombinant TGF-β1 reduced BBB permeability, however its effects on tight junction integrity or oedema associated with inflammatory pain were not investigated [74].

Recent studies have also highlighted the potential link between endothelial dysfunction and migraine. Migraine is associated with elevated serum levels of claudin-5 [145]. However in a KCl-induced model of episodic headache, no changes in tight junctions were observed despite increased BBB permeability [146].

### Therapeutic targeting of claudin-5

Therapeutic targeting of claudin-5 can take the form of transiently downregulating expression of the protein for a temporary and reversible size-selective increase in BBB permeability or by targeting upstream signalling components that regulate claudin-5 expression. RNA interference has been utilised in models of traumatic brain injury, Alzheimer’s disease, and choroidal neovascularisation to either remove unwanted, neurotoxic material from the brain or to increase the CNS penetration of small molecule therapeutics for improved efficacy [57, 119, 147, 148]. Other techniques that have been used to specifically target claudin-5 include peptides targeting the ECLs of claudin-5. Based on this approach, it is possible to increase the size-exclusion limits of the BBB by selectively downregulating claudin-5. This approach facilitated enhanced penetration of the chemotherapy doxorubicin (580 Da) in vivo while increasing permeation of molecules up to 40 kDa in vitro [149]. These approaches are of considerable interest given the transient nature of claudin-5 downregulation and BBB modulation with levels of claudin-5 and BBB integrity returning to normal within 24 h. As mentioned in previous sections, circulating peripheral blood leukocytes were found to express claudin-5, likely derived from extracellular vesicles released by the capillary endothelium or by physical contact of leukocytes with the endothelium. In multiple sclerosis patients, treatment with glucocorticoid lowered mRNA and protein levels of claudin-5 and a significant correlation was found between increased levels of leukocyte claudin-5 protein and multiple sclerosis disease activity [102]. Glucocorticoid treatment can offer BBB protection. Progesterone and allopregnanolone reduce neuroinflammation and improve barrier function in a mouse model of ischemic stroke by downregulating MMP expression and preventing degradation of claudin-5 and occludin [150].

Outside of the CNS, claudin-5 dysfunction is implicated in numerous disorders (Table 1). Claudin-5 is severely downregulated following heart failure in post-mortem heart samples [33]. In a mouse model of cardiomyopathy and muscular dystrophy, claudin-5 levels are drastically reduced. Restoration of claudin-5 levels prevented the physiological hallmarks of cardiomyopathy and improved histological outcome. This was achieved following a single administration of a recombinant adeno-associated viral vector (AAV6) containing a mouse claudin-5 expression cassette under the control of the minimal cytomegalovirus promoter to the hearts of mice with cardiomyopathy [34]. These studies highlight the therapeutic potential of restoring junctional integrity by overexpressing claudin-5.

## Concluding remarks

Tight junctions are a vital component of the brain endothelium, regulating the exchange of material between blood and brain and protecting delicate neural tissue from damaging blood-borne agents such as pathogens and immune cells. This review focussed on the role of claudin-5 in maintaining paracellular integrity and its regulation in homeostatic and pathological scenarios. Claudin-5 is a highly enriched tight junction protein vital to the physical barrier properties of the BBB with its loss or disruption acutely involved in neurodegenerative, neuroinflammatory and neuropsychiatric disorders. Various upstream signalling components can regulate claudin-5 levels and manipulating these pathways to modulate claudin-5 expression in response to disease is a viable therapeutic strategy. In addition, RNAi-mediated suppression of claudin-5 is a well characterised strategy to temporarily modulate BBB permeability to low molecular weight therapies in preclinical models of disease which may be useful for treating various CNS disorders. Downregulation of claudin-5 is an early and prominent feature of MS and epilepsy, suggesting that early loss of claudin-5 and breakdown of paracellular BBB integrity is an important feature of the pathogenesis of these disorders. Strategies to restore tight junction integrity should be investigated. From a functional perspective, we still do not understand all of the regulatory molecules governing claudin-5 fate. In addition, we lack a crystal structure of claudin-5 and how exactly it interacts to form tight junction strands. Furthermore, there is little understanding of the additional roles of claudin-5 beyond regulating paracellular permeability. A comprehensive understanding of claudin-5 based tight junctions and the biological systems they control will lead to novel therapeutic targets for various diseases.
